# Evaluating the ergonomic aspects of laparoscopic energy devices: combination of a survey and a kinesiologic experiment

**DOI:** 10.1007/s00464-025-12082-9

**Published:** 2025-09-09

**Authors:** Hee Ju Sohn, Junkyung Song, Yoo Shin Choi, Youngmin Han, Wooil Kwon, Jaebum Park, Jin-Young Jang

**Affiliations:** 1 Department of Surgery and Cancer Research Institute, Seoul National University Hospital, Seoul National University College of Medicine, Seoul, Korea; 2 Department of Surgery, College of Medicine, Chung-Ang University, Seoul, Korea; 3 Department of Physical Education, Institute of Sports Science, Advanced Institute of Convergence Science, Seoul National University, 71-1, #414, 1 Gwanak-Ro, Gwanak-Gu, Seoul, 08826 South Korea

**Keywords:** Laparoscopic surgery, Energy devices, Ergonomics, Muscle fatigue

## Abstract

**Introduction:**

This study aimed to evaluate surgeons’ ergonomic satisfaction when using laparoscopic energy devices and to investigate how prolonged use affects muscle fatigue and surgical performance.

**Methods:**

A two-part study, including a survey and a kinesiologic experiment, was conducted to compare 4 laparoscopic energy devices (D1–D4). Thirty surgeons completed a structured survey assessing ergonomic factors such as device weight, grip strength, handle design, comfort, and trigger location. In parallel, a kinesiologic experiment involving 20 surgeons measured device activation force, surface electromyography (EMG) changes, and targeting accuracy before and after fatigue induction using a standardized protocol.

**Results:**

The survey revealed significant ergonomic differences among devices. In the kinesiologic study, two devices showed a significant decline in precision and targeting accuracy after fatigue. All but one device demonstrated significant changes in the median frequency (MDF) and integrated EMG (iEMG) of the flexor digitorum superficialis muscle (*P* < 0.001), indicating muscle fatigue.

**Conclusion:**

Prolonged use of certain laparoscopic energy devices induces muscle fatigue and impairs accuracy. These findings underscore the importance of ergonomic considerations in surgical instrument design to enhance surgeon performance and reduce fatigue-related risks.

**Supplementary Information:**

The online version contains supplementary material available at 10.1007/s00464-025-12082-9.

In recent years, there has been substantial growth in laparoscopic surgery because of its capacity to reduce postoperative pain and facilitate enhanced recovery compared to open surgery [1, 2]. Since the advent of laparoscopic surgery, there have been marked improvements in surgical techniques, the development of innovative surgical devices, and advancements in the surgical environment. However, due to the nature of laparoscopic surgery, which requires surgeons to maintain a fixed posture for extended operative times, higher levels of physical and mental stress are accompanied [3–5].

Surgeons who actively perform a large number of laparoscopic procedures often suffer from musculoskeletal disorders involving the wrists, shoulders, and lower back [3, 6, 7]. Moreover, these specific discomforts have been associated with the use of certain devices, which can vary based on factors such as hand size and sex [8, 9]. For instance, operating heavy devices with large handles can be particularly uncomfortable for surgeons with small hands or for female surgeons. Yet most surgical devices are released in a single standard size. A recent study by Kono et al. reported sex-based disparities in ergonomic fit, emphasizing the need for handle redesign to improve gender equity in surgical tools [10].

Various laparoscopic energy devices are available on the market, each with a specific design and operating mechanism. Surgeons make their selections based on the device’s hemostatic or dissecting capability and tissue compatibility. Some devices have been reported to cause discomfort during use. Most previous studies on laparoscopic energy devices have focused on aspects such as hemostatic efficacy and performance of the device itself [11] or on surgical outcome [12]. In contrast, studies evaluating the ergonomic features of these devices in actual use are limited.

The primary objective of this study was to discern the ergonomic challenges faced by surgeons while using surgical energy devices and to evaluate the impact of prolonged use of these devices on surgeons’ ergonomic strain and surgical performance. The ultimate goal is to improve the surgical environment by optimizing the ergonomic design of laparoscopic energy devices, eventually enhancing the quality and safety of surgeries.

## Material and methods

### Study design and participants

**Fig. 1:**
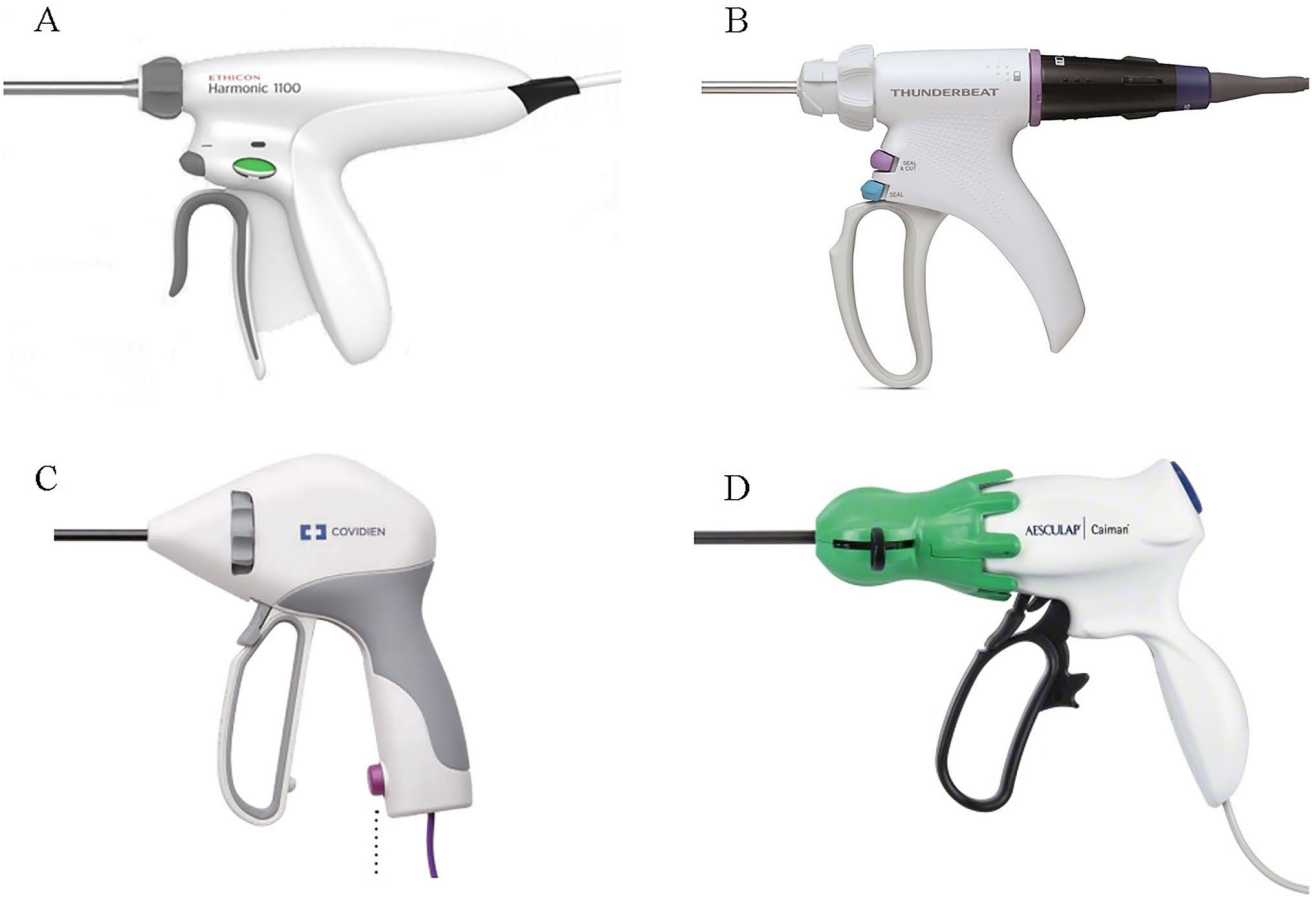
Handle designs of four laparoscopic energy devices (D1-D4). **A** D1; HARMONIC^®^, Ethicon. **B** D2; THUNDERBEAT, Olympus. **C** LigaSure^™^, Medtronic. **D** Caiman^®^ 5, Aesculap

The current study is composed of two components: (1) a survey given to surgeons actively involved in leading or assisting surgical procedures and (2) a kinesiologic experiment with the same participants to evaluate four laparoscopic energy devices. The devices under investigation were HARMONIC^®^ HD 1000i Shears (Ethicon Endo-Surgery, Inc. Cincinnati, OH, USA) labeled D1, THUNDERBEAT (Olympus, Tokyo, Japan) labeled D2, LigaSure^™^ Maryland Jaw (Medtronic, Minneapolis, MN, USA) labeled D3, and Caiman^®^ 5 (Aesculap Inc., Center Valley, PA, USA) labeled D4 (Fig. 1). Thirty surgeons participated in the survey and 20 surgeons participated in the kinesiology experiment.

Surgeons were recruited from two tertiary hospitals, Seoul National University Hospital and Chung-Ang University Hospital, and included residents, fellows, and staff specializing in the surgery department. Individuals with a history of surgery and those currently on medication for upper extremity or spine-related disorders were excluded.

### Survey

The administered questionnaire (Supplement 1) aimed to evaluate the subjective experiences and ergonomic concerns regarding device use. The survey was conducted by allowing participants to physically handle and compare the four devices in front of them. The basic survey items included age, sex, dominant hand, glove size, level of experience in laparoscopic surgery, and a history of musculoskeletal disorders.

The specific survey items included nine different ergonomic factors: appropriateness of the device’s weight, force required to grip, force required to maintain the clamp, suitability of the handle size, comfort of the handle design, appropriateness of the force required to activate the trigger, location of the activation trigger, ease of operating the rotation knob, and the overall ergonomic score.

### Kinesiologic experiment

The experiment used a repeated-measures design, with four laparoscopic energy devices evaluated by the same participants. Each participant visited the laboratory four times to test each of the four devices. Baseline grip force was measured using a calibrated dynamometer prior to each experiment. This measurement ensured that participants began each session with comparable muscle activation levels, which could otherwise influence their perception and performance during the evaluation of the devices. Grip force variability was required to remain within 10% of the participant’s initial baseline (measured during their first session) to account for natural fluctuations in muscle strength due to fatigue, physical condition, or other factors. If the variation exceeded this threshold, the experiment was postponed or the participant was excluded to maintain consistency and reliability in the results.

**Fig. 2:**
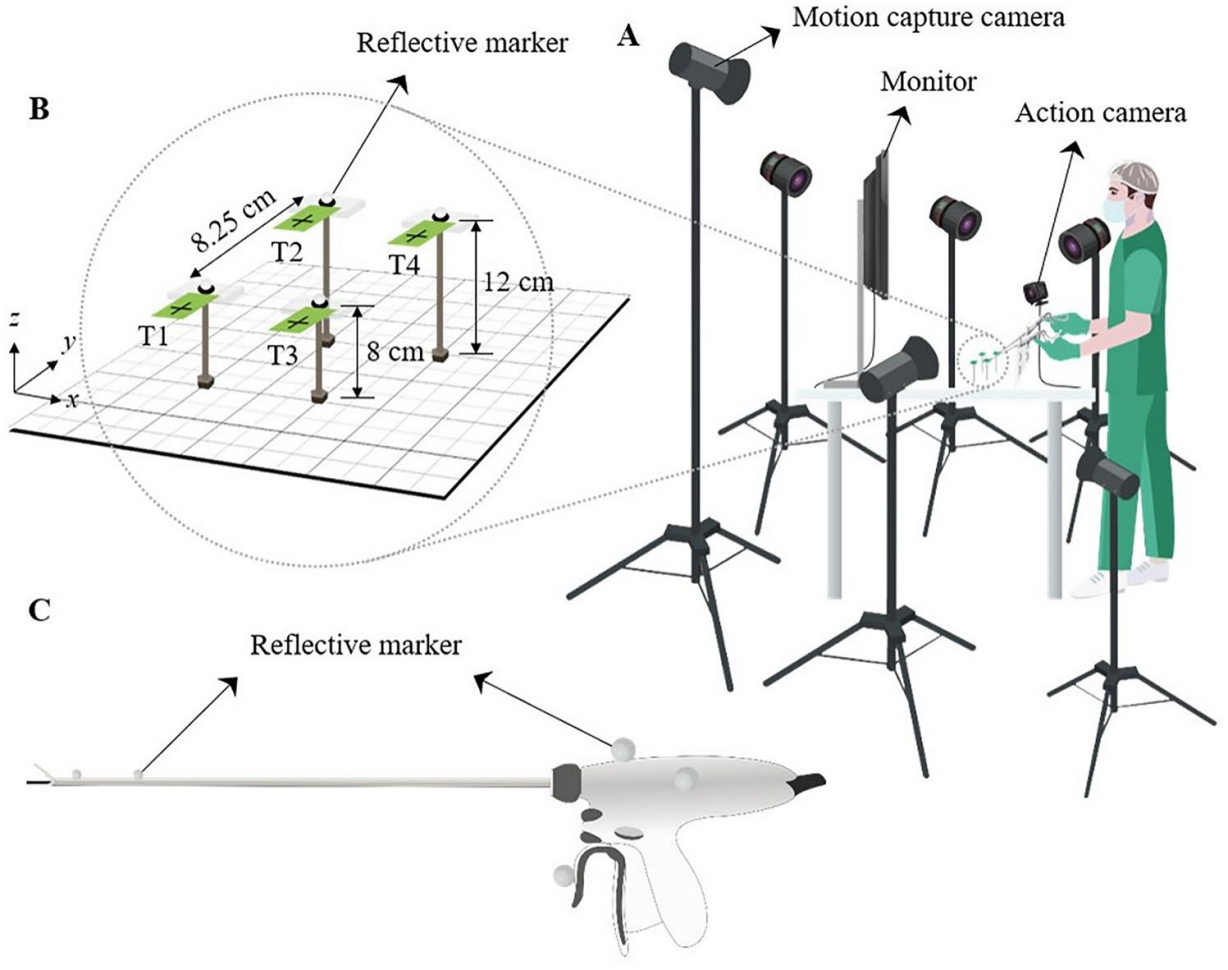
Schematic diagram of experimental setup **A** represents a schematic of the setting used by the participant during the experiment, and **B** shows an enlarged view of the targets used during the experiment. The targets were placed at a fixed distance from each other and the participants were instructed to manipulate them in the order T1 > T2 > T3 > T4. **C** indicates the markers attached to the instrument, which are used to gather data in the Motion Capture System

A customized targeting system, similar to a laparoscopic training simulator, was developed. The system had four targets (T1, T2, T3, and T4, as shown in Fig. 2B) on a square base (Fig. 2C), which the participants could aim at and grasp using the test device. The targets were arranged in a square configuration, with each target separated by 8.25 cm. Two targets (T1 and T2) were positioned at a height of 8 cm, while the other two targets (T3 and T4) were at a height of 12 cm above the base. Real-time feedback on the target and device positioning was provided by using an action camera linked to a monitor. Spherical reflective markers were affixed to the energy devices and atop each target (Fig. 2). The dynamic positions of each marker were recorded at 100 Hz using a motion capture system with six infrared cameras (Prime 13, OptiTrack, Natural Point Inc., Corvallis, OR, USA).

Each participant performed the target task on a separate day for each device. The task consisted of six cycles. Each cycle required participants to navigate sequentially from T1 to T4. The participants were instructed to move the tip of the device to the designated target for 3 s and grasp the cross-marked center for 5 s as precisely as possible. Throughout the task, wireless EMG equipment recorded the electrical activity of the right upper limb muscles (Trigno Wireless EMG System, Delsys Inc., Natick, MA, USA). After each cycle, the participants performed forearm exercises for muscle fatigue loading with a hand gripper for 3 min, synchronized with metronome signals. Eight electrodes were placed on the flexor digitorum superficialis (FDS), extensor digitorum (ED), flexor carpi radialis (FCR), extensor carpi radialis (ECR), biceps brachii (BB), triceps brachii (TB), middle deltoid (DEL), and upper trapezius (UTP). The study adhered to the SENIAM (Surface Electromyography for the Non-Invasive Assessment of Muscles) guidelines, recognized as the gold standard for EMG electrode placement. A trained kinesiologist applied the surface EMG electrodes to all participants to ensure consistency across sessions.

### Outcomes: EMG data and motion accuracy and precision

EMG data were analyzed to determine integrated EMG (iEMG) and median frequency (MDF). The iEMG signals, which signify muscle activation intensity and fatigue, were derived from these integrated signals. Each participant’s iEMG for every target and cycle was normalized against the peak iEMG observed during four repeated measurements. The filtered EMG signals were then windowed and transformed into a power spectrum using a fast Fourier transformation.

To assess the targeting performance influenced by muscle fatigue, the target accuracy index (ACI) and precision index (PRI) were calculated based on the marker positions on the targets. ACI represents the Euclidean distance between the center of the cross-marked target and the point at which the tip of the device landed. A higher ACI value indicates a lower accuracy in grasping the target. The PRI quantifies the stability of the grasp, measured as the square root of the difference in Euclidean distances between the average of the device tip. This index shows the deviation magnitude due to the device tip tremors while holding the target.

### Statistical analysis

One-way analysis of variance (ANOVA) was conducted to compare the means of the four devices when analyzing the survey results. The assumptions of normality and homogeneity of variances were verified. A post-hoc analysis using Tukey’s test was performed to identify specific group differences.

To evaluate the effect of muscle fatigue across different laparoscopic energy devices, we conducted a separate device × fatigue repeated-measures ANOVA for maximal grip strength and the set of kinesiologic and EMG variables. The Device factor includes four different energy devices, and the fatigue factor includes two levels: pre-fatigue (PRE) and post-fatigue (POST), corresponding to the initial and final cycles. Additionally, regression analyses were performed to discern the patterns of change (e.g., linear increase or decrease) in the dependent variables over the six cycles owing to accumulating fatigue. Correlation analysis [13] was further employed to assess the relationship between kinematic outcomes (ACI and PRI) and fatigue index change (MDF). Mauchly’s test ensured sphericity assumptions, with violations adjusted using the Greenhouse–Geisser estimate. We conducted all pairwise comparisons with Bonferroni adjustments to explore significant interactions and main effects. The effect size is represented as partial eta squared (ηp^2). All statistical evaluations were performed using SPSS Statistics (version 24.0; IBM Corp, Armonk, NY, USA), with the significance threshold set at *P* < 0.05.

## Results

### Survey

A total of 30 participants took part in the survey, consisting of 15 males and 15 females. The participants included 22 residents and 8 faculty members working in the surgery department. The average age was 37.5 years, and all participants were actively performing or assisting in laparoscopic surgeries. While some participants mentioned that they had not used certain instruments among the four provided, this survey focused on their intuitive perceptions rather than performance during surgery, which is not expected to pose a significant issue. The survey results of ergonomic score are presented in Table 1. Although each of the evaluated devices was actively used in surgeries, statistically significant differences were noted in all the evaluated aspects.

**Table 1:** Results of questionnaire of the actual use (10-point scale)

	D1	D2	D3	D4	p	f	Post-hoc
Weight	7.3	6.9	8.2	7.5	0.012	3.779	D3 > D2
Clamp force	8.3	6.7	6.7	4.6	< 0.001	20.130	D1 > D3,4 > D2
Clamp maintenance force	8.0	6.3	5.8	7.6	< 0.001	6.621	D1 > D2,3
Handle size	8.2	7.5	7	6	< 0.001	10.731	D1 > D3,4
Handle comfort	8.5	7.8	7.1	5.5	< 0.001	16.807	D1 > D3 > D4
Activation trigger (force)	8.2	7.7	6.1	5.4	< 0.001	16.999	D1,2 > D3,4
Activation trigger (location)	7.9	7.9	7.7	5.6	< 0.001	11.372	D1,2,3 > D4
Rotation knob	7.8	6.6	6.3	6.9	0.026	3.196	D1 > D3
Overall ergonomic comfortability	8.3	7.1	7	6.1	< 0.001	15.509	D1 > D2,3 > D4

D1: HARMONIC HD 1000i Shears, D2: THUNDERBEAT, D3: LigaSure Maryland jaw, D4: Caiman 5

D3 received the highest weight score, indicating that it was perceived as having the most appropriate weight among the devices, whereas D2 scored the lowest in this category (8.2 vs. 6.9; *P* = 0.001). D1 received the highest score for clamp force (8.3), significantly outperforming D4 and D2 (4.6 and 6.7, respectively; *P* < 0.001). For clamp maintenance force, D1 had a higher score than D2 and D3 (8.0 vs. 6.3 vs. 5.8, respectively; *P* < 0.001). D1 had the best score for handle size (8.2), indicating superior comfort and suitability for size, whereas D3 (7.0) and D4 (6.0) scored significantly lower (*P* < 0.001).

For the force required to activate the trigger, D1 and D2 scored high, at 8.2 and 7.7, respectively, while D3 and D4 scored lower, at 6.1 and 5.4, respectively. In the post-hoc analysis, the D1 and D2 scores were significantly higher than those of D3 and D4 (*P* < 0.001). Both D1 and D2 scored the highest for trigger location at 7.9, followed closely by D3 (7.7), and D4 scored the lowest at 5.6 (*P* < 0.001). Many participants reported discomfort with the trigger location on D4 and mentioned that it took a considerable amount of time to adapt to using it during regular surgeries. Although the rotation knob is typically located at a similar position across devices and is primarily operated with the index finger, some devices have been found to be uncomfortable for individuals with smaller hands or shorter fingers. In this study, D1 scored the highest in this category at 7.8 and D3 scored the lowest (*P* = 0.026). Regarding the overall ergonomic comfort, D1 had the best score of 8.3, followed by D2 (7.1), D3 (7.0), and D4 (6.1) (*P* < 0.001). In the post-hoc analysis, the score for D1 was significantly higher than that for D2 and D3, which in turn was higher than that for D4.

We further examined sex differences in survey responses. For D1, men rated the weight as appropriate and assigned high scores, while women assigned significantly lower scores (8.0 vs. 6.7, *P* = 0.048). For D4, the trigger activation force received significantly lower scores in women than in men, indicating a higher level of discomfort (6.3 vs. 4.5, *P* = 0.024).

### EMG data

The maximal voluntary contraction (MVC) of each participant was measured before and after the task, using a digital handgrip dynamometer. We observed a significant decrease in the MVC for all devices (Supplement 2). This consistent decrease among all the participants validated the occurrence of muscle fatigue in the forearm during the experiment. No notable differences in the MVC reduction were observed across the devices. The initial trial (without muscle fatigue, PRE) was set up in contrast to the sixth trial (with muscle fatigue, POST).

**Fig. 3:**
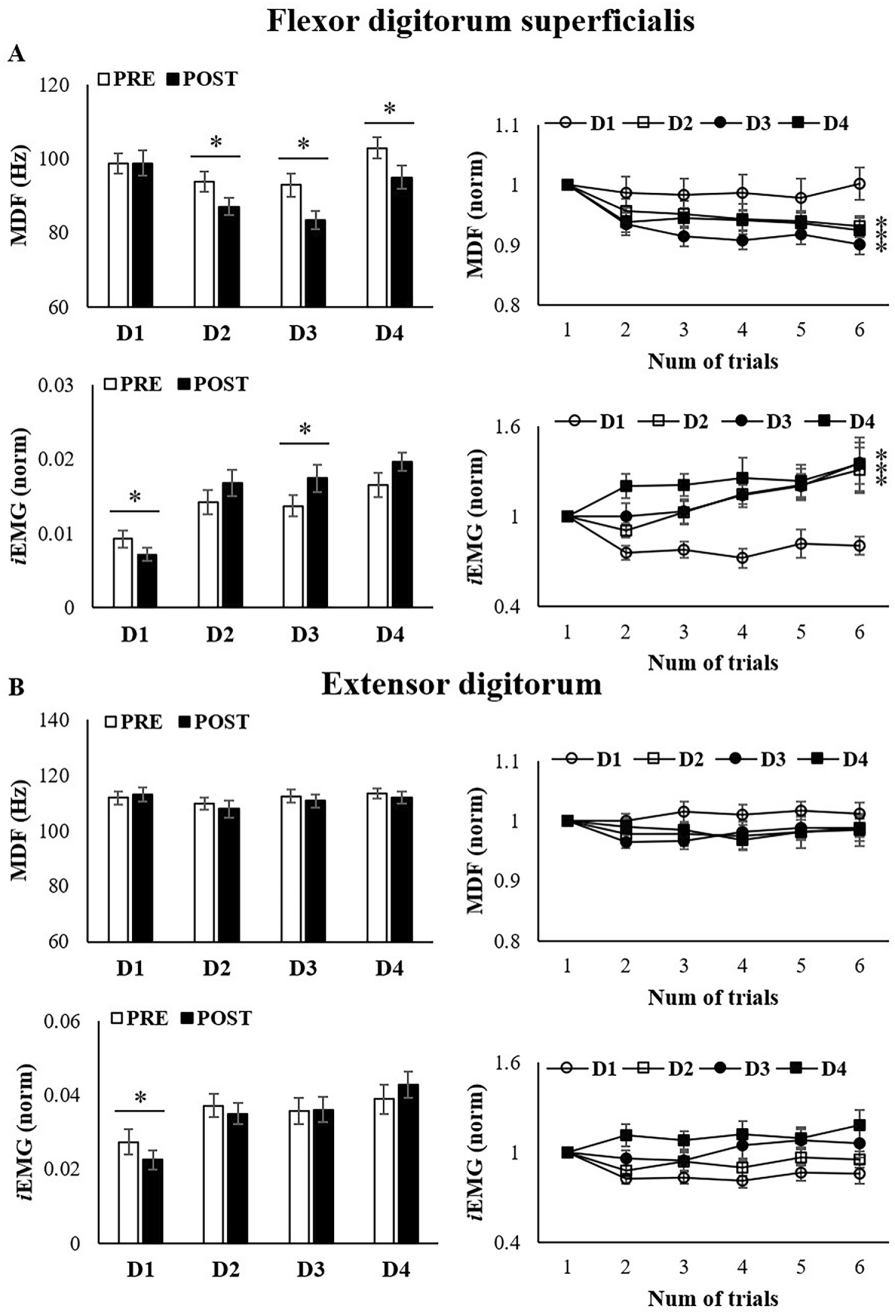
Results of EMG profile among 4 laparoscopic energy devices The median frequency (MDF) indicates a point in the frequency spectrum of the EMG signal that divides the power spectrum into two equal halves. Integrated electromyography (iEMG) refers to the process of integrating the raw EMG signal over a period of time, which reflects muscle strength and tension. (**A** Results for flexor digitorum superficialis. **B** Results for extensor digitorum)

Figure 3 presents the EMG results for the FDS muscle, which is the most active muscle during device use, and its antagonist, the ED muscle. MDF served as a muscle fatigue index, indicating a downward frequency spectrum shift. Significant MDF changes were found before PRE and POST for D2 (*P* = 0.001), D3 (*P* < 0.001), and D4 (*P* = 0.002). Linear regression analyses revealed significant changes in MDF values for D2 (R^2^ = 0.075, *P* = 0.009), D3 (R^2^ = 0.169, *P* < 0.001), and D4 (R^2^ = 0.085, *P* = 0.005). However, there were no significant EMG changes on D1, indicating that this device induces low-level fatigue.

iEMG quantifies total electrical activity over a given time, signaling muscle tension and fatigue. In the PRE-POST paired sample test, we found a decrease in iEMG values on D1 (*P* = 0.007) and a significant increase on D3 (*P* = 0.012). When comparing the devices, the iEMG values for D1 were lower than those for D2, D3, and D4, suggesting less post-muscle fatigue. Linear regression indicated increasing iEMG trends during tasks D2 (R^2^ = 0.118, *P* = 0.001), D3 (R^2^ = 0.126, *P* = 0.001), and D4 (R^2^ = 0.047, *P* = 0.040). However, the iEMG values for the ED muscle revealed no significant differences in either PRE-POST or between devices.

Data for other muscles are presented in Supplement 3. Significant differences in the FCR, DEL, and UTP were observed between the devices after inducing fatigue. In the comparison between devices, it was observed that in the FCR, D4 had higher iEMG values than the other devices, indicating an increased ergonomic strain during prolonged surgeries. In the DEL and UTP, which reflect fatigue in the shoulders and back, respectively, a significant increase in EMG values was observed across all devices; however, no differences were observed among them.

### Kinesiologic data

**Fig. 4:**
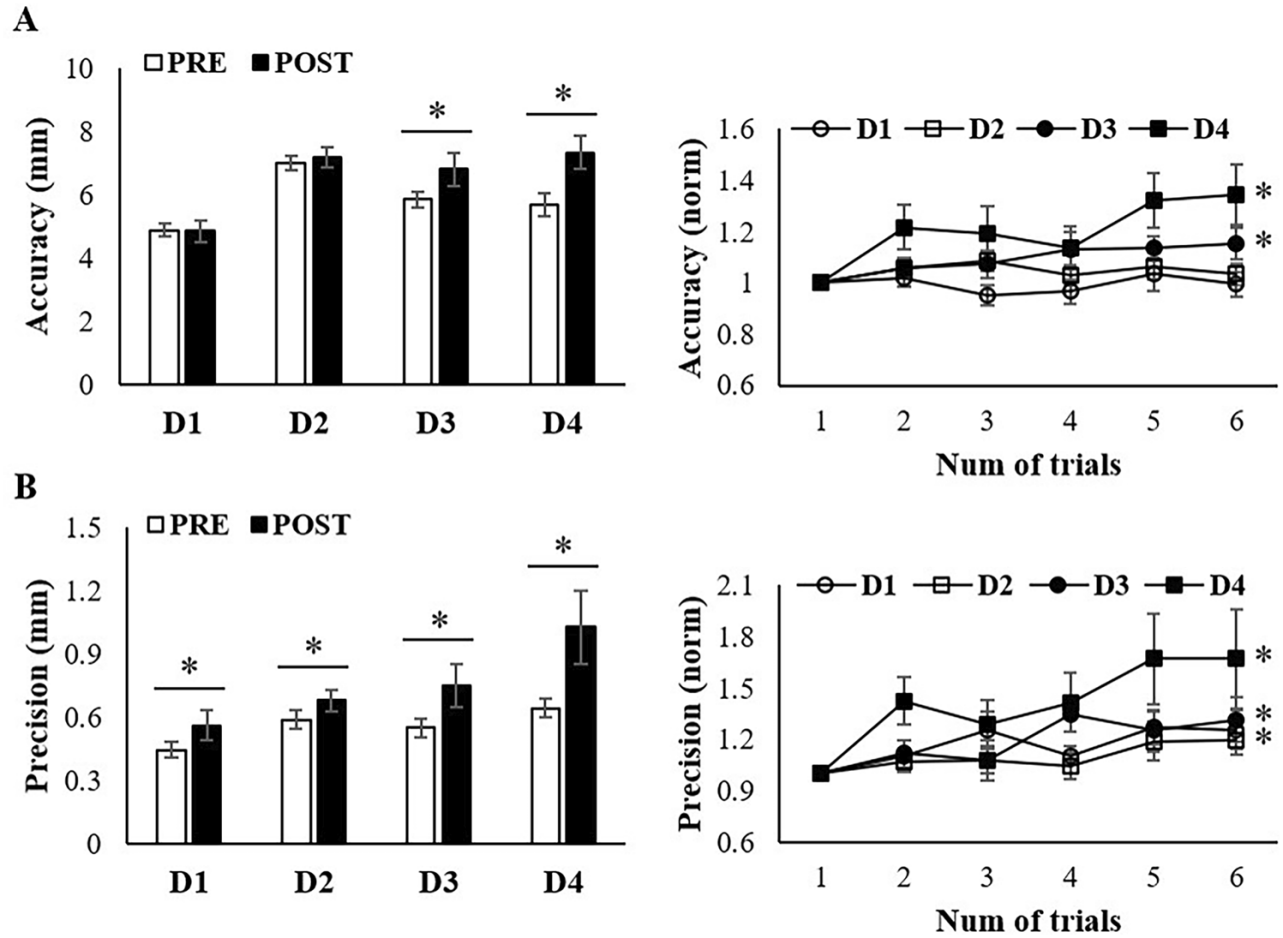
Results of kinematic data of accuracy and precision. The left graph presents the results of a 2-way ANOVA performed using data from the first trial, where fatigue had not been set, and the last and sixth trials. The right graph displays the data normalized to the values from the first trial to observe the pattern of change in the dependent variable due to the onset of fatigue. The asterisk (*) indicates a device that shows significant results based on linear regression

Figure 4 shows the accuracy and precision values for the four devices, as well as the PRE-POST fatigue levels, measured as the distance from the target point using a motion capture system. A lower value suggested greater accuracy and precision. Notable accuracy deviations between the PRE and POST) were detected for D3 and D4. In the non-fatigued state, the scores for D3 and D4 were lower than those for D1 and D2 (D1 < D2, D3; D2 < D3, D4). When comparing the ACI pre- and post-fatigue, significant differences emerged for D3 and D4, whereas D1 and D2 remained unchanged (D3: R^2^ = 0.072, *P* = 0.010 and D4: R^2^ = 0.069, *P* = 0.013). Among the four devices, D1 and D2, particularly D1, exhibit the best ACI results.

All devices exhibited PCI changes according to the presence of fatigue (D1, p = 0.037; D2, *P* = 0.045; D3, *P* = 0.026; D4, *P* = 0.048), and significant differences in PCI were observed between D1 and D4 before and after fatigue (PRE, *P* = 0.007; POST, *P* = 0.023). D1 had superior results in terms of precision compared to D4. Linear regression analysis revealed significant trends in D1 (R^2^ = 0.055, p = 0.026), D3 (R^2^ = 0.083, p = 0.006), and D4 (R^2^ = 0.074, p = 0.009).

### Correlation between EMG and kinesiologic data

**Fig. 5:**
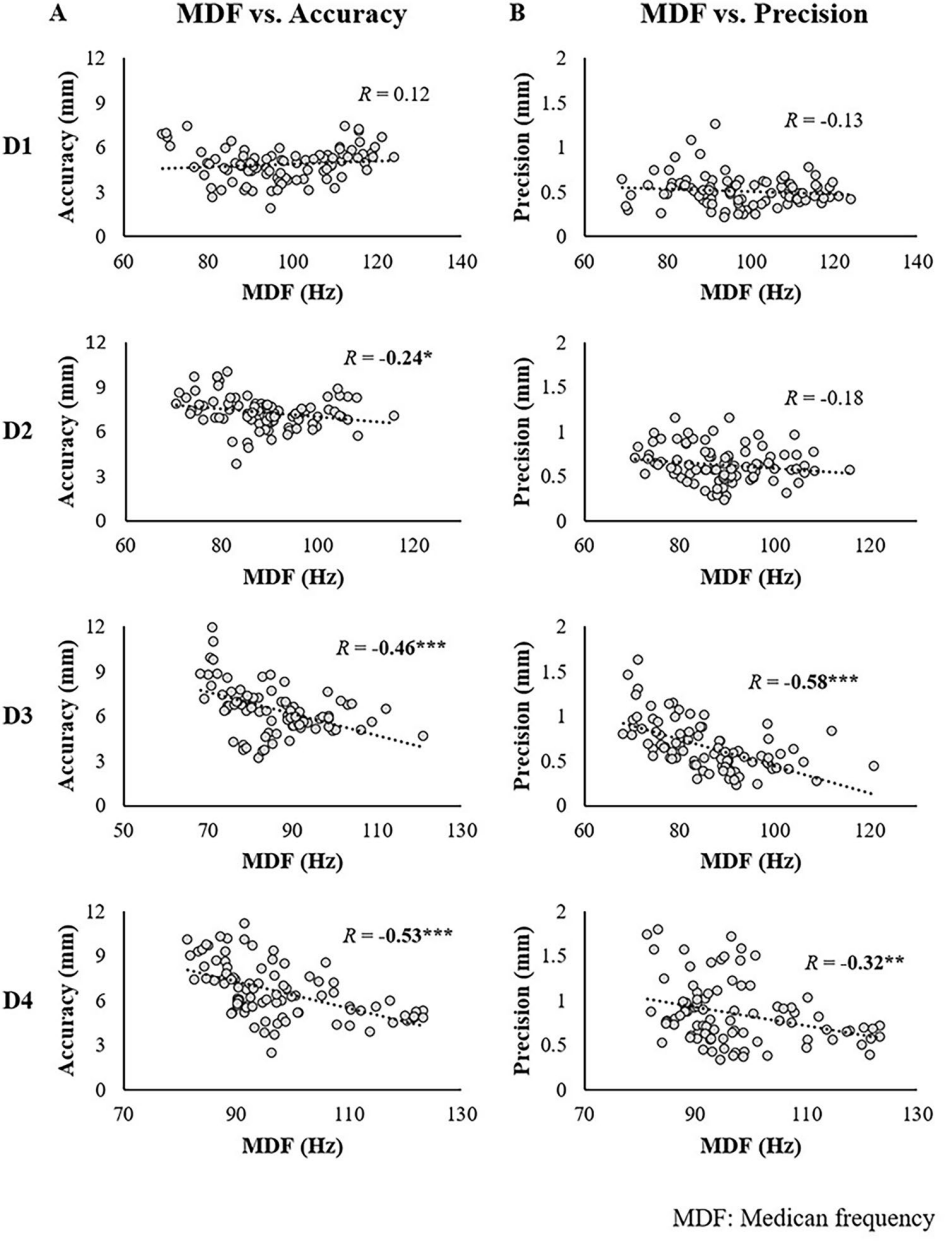
Relationship between median frequency (MDF) versus kinematics. The correlation analysis results between the median frequency and kinematics (Accuracy, Precision) showed a moderate association with the accuracy (R = -0.46) and precision (*R* =  − 0.58) of D3, as well as the accuracy (*R* =  − 0.53) of D4

The relationship between the changes in MDF and the kinetic parameters for each device is summarized in Fig. 5. A moderate negative correlation between MDF and kinetic parameters was observed for D3, with R-values of − 0.46 for MDF vs. accuracy and − 0.58 for MDF vs. precision. A moderate correlation between MDF and precision (*R* =  − 0.53) was also found for D4, whereas no significant relationship between MDF and kinetic parameters was detected for D1 and D2.

## Discussion

Surgeons typically select the energy device to use based on the type of surgery (considering the organ and tissue involved), the device’s hemostatic and dissecting capabilities, and specific features. Device versatility is high, particularly in laparoscopic surgery, where its use is multidimensional and encompasses tasks, such as tissue dissection, vessel ligation, and hemostasis. Hence, convenience of use is considered a critical factor. In our survey, individuals with smaller hands, lower muscle strength, and female doctors found it quite challenging to use some of the devices. Moreover, despite the gradual increase in the number of female doctors, surgical devices are still predominantly produced in a single standard size [14]. These findings suggest that sex-based anatomical differences, such as smaller hand size and grip strength, may contribute to ergonomic discomfort. As most devices are not tailored to diverse users, female surgeons may be disproportionately affected. This underscores the need for more inclusive instrument design that reflects the growing diversity of the surgical workforce.

The EMG and kinesiology data obtained in the current study were analyzed in collaboration with clinicians and kinesiology experts. A significant effort was made to set up an experiment that closely mimicked the actual surgical environment. Various factors were controlled to accommodate the unique characteristics of the devices and investigate the fatigue factor. The desk height and position of the monitor were customized according to the height and arm position of the participants. Those unfamiliar with the four devices were given sufficient time to practice and familiarize themselves with the study protocols. Several pilot tests were conducted before the design of the main experiment.

Notably, differences in accuracy and precision were observed, even in the initial trial of the experiment without fatigue. Additionally, fewer EMG changes were observed for D1 than for the other devices, even under fatigue conditions, indicating resilience to fatigue with the use of this device. This indirectly demonstrates that D1 has a more ergonomic design and superior user convenience compared with the other evaluated devices. In the correlation analysis between EMG and kinematic data, bipolar energy devices (D3 and D4) generally showed stronger correlations, suggesting that larger fluctuations in EMG activity are associated with increased position errors. Additionally, in the survey results, scores for handle size and trigger activation force demand were lower for the bipolar energy device, indicating that devices that cause subjective discomfort could also affect the quantitative EMG results and accuracy. In cases where the surgery time is long, these effects could have a greater impact on the accuracy for surgeons with lower basic muscle strength [15].

Prior studies detailing the process of developing an ergonomic design for a new laparoscopic grasper [16–18] have confirmed the tool’s efficacy and user satisfaction, contributing significantly to advancements in the field. Rodriguez et al. [19] and Zihni et al. [20] reported higher muscle activation in laparoscopic surgery than in robotic surgery during several tasks in a laboratory setting. This suggests that robotic surgery may be more ergonomically favorable in certain conditions.

Studies conducted on live surgery [21–23] have further highlighted the ergonomic advantages of robotic surgery. These studies reported that robotic surgery causes less ergonomic strain than laparoscopic surgery on certain muscles [24], likely due to improved instrument control and better posture maintenance facilitated by robotic systems. However, it is important to consider that mental stress factors might negate some of these ergonomic benefits. One study suggested that if mental stress factors are considered, robotic surgery might result in even greater ergonomic strain [25]. Furthermore, factors such as surgical experience [26], role during surgery (chief surgeon vs. assistant surgeon) [27] and smaller hand size [28] also influence the physical workload.

A major strength of this study is that we presented objective data through collaborative research with ergonomic experts. Through preliminary experiments, we made the best efforts to minimize errors. To ensure that the data is accepted by all surgeons, it is important to highlight the multidisciplinary nature of our study. The subjects of the experiments were surgeons, and the experimental design, monitoring during the experiments, and data analysis were conducted in collaboration with experts in ergonomics.

Nonetheless, some bias may exist due to varying levels of laparoscopic experience among participants. As the study was conducted in an experimental setting, it may not fully reflect the complexity and stress of real surgical environments. As a result, the muscle overload induced in the experiment may have been somewhat exaggerated and the tremors are also included in the results. Furthermore, sex differences and hand size variations, which are important factors, were not included in this study to focus on comparisons between pre- and post-fatigue conditions and among the devices. These variables will be addressed in future research. In addition, the relatively small sample size in the kinesiologic experiment (*n* = 20) may have limited the statistical power. Finally, all participants in this study were Koreans, and the devices may not be equally suitable for larger European or American populations.

Considering that the devices under study may soon be replaced with newer devices or platforms, innovations such as virtual reality [29], augmented reality, and advanced simulation training systems hold promise for improving surgical ergonomics. In addition, future research should aim to incorporate a more detailed comparison between laparoscopic and robotic surgeries, considering both physical and mental ergonomic factors. By doing so, we can better understand the comprehensive impact of these surgical methods and develop interventions that optimize surgeon performance and well-being.

In summary, we found notable differences in subjective satisfaction across devices, depending on the user. During prolonged use, some devices had less impact on muscle fatigue, whereas others induced variance in performance metrics such as accuracy. More ergonomic tools will undoubtedly lead to safer surgeries and ultimately benefit patients. Therefore, surgeons should play an active role and provide feedback on ergonomics, thus aiding the development of improved devices and better surgical outcomes.

## Supplementary Information

Below is the link to the electronic supplementary material.

Supplementary file1 (JPG 260 KB)

Supplementary file2 (JPG 48 KB)

Supplementary file3 (JPG 193 KB)
