# Supplementary figures and images for: Evaluating the ergonomic aspects of laparoscopic energy devices: combination of a survey and a kinesiologic experiment
Source: Surg Endosc. 2025 Sep 9;39(11):7576–85. doi: 10.1007/s00464-025-12082-9 (PMC12618446; doi:10.1007/s00464-025-12082-9)

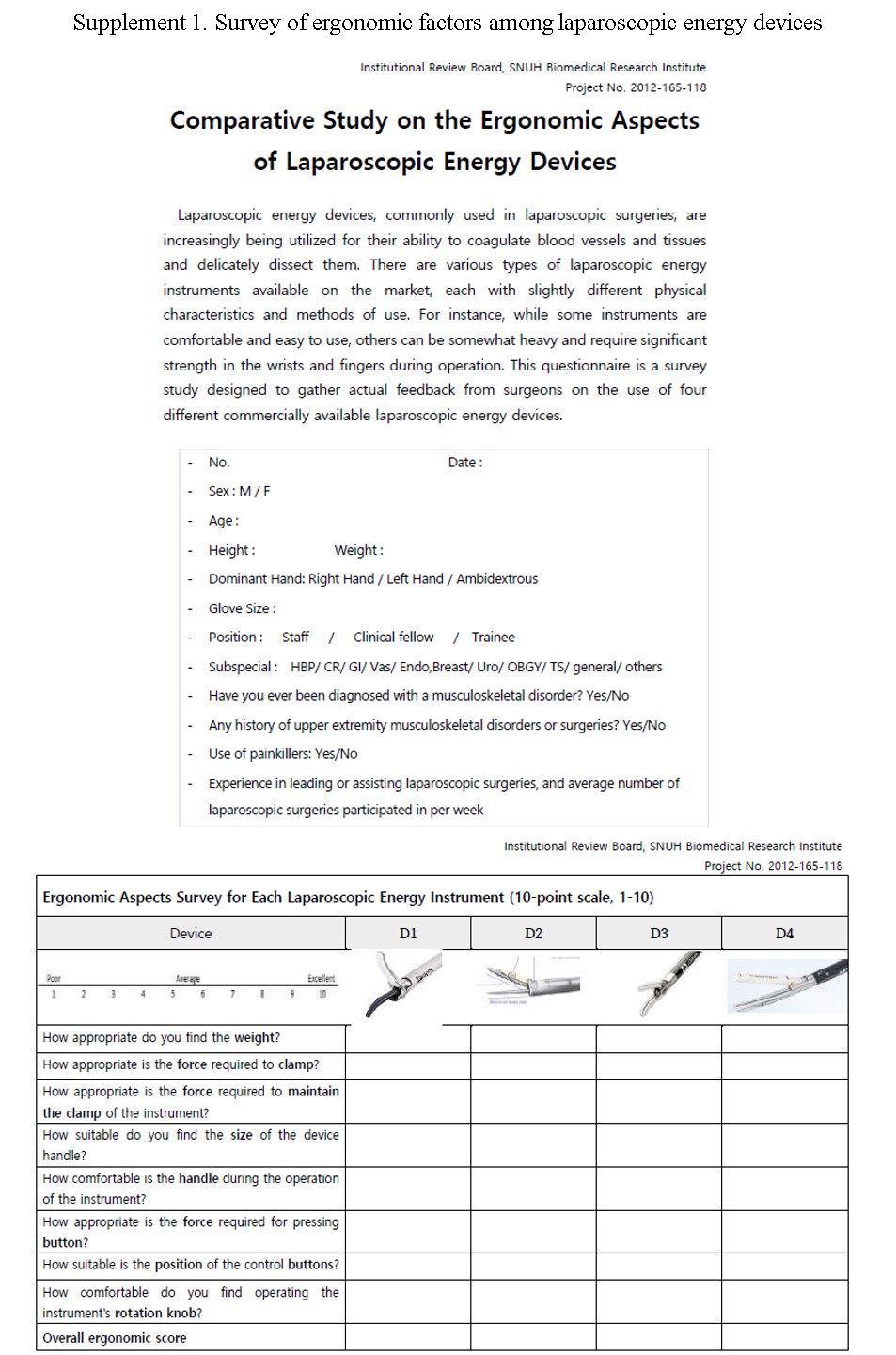

Supplement: Supplementary file 1 — Supplementary file1 (JPG 260 KB) [file 464_2025_12082_MOESM1_ESM.jpg]

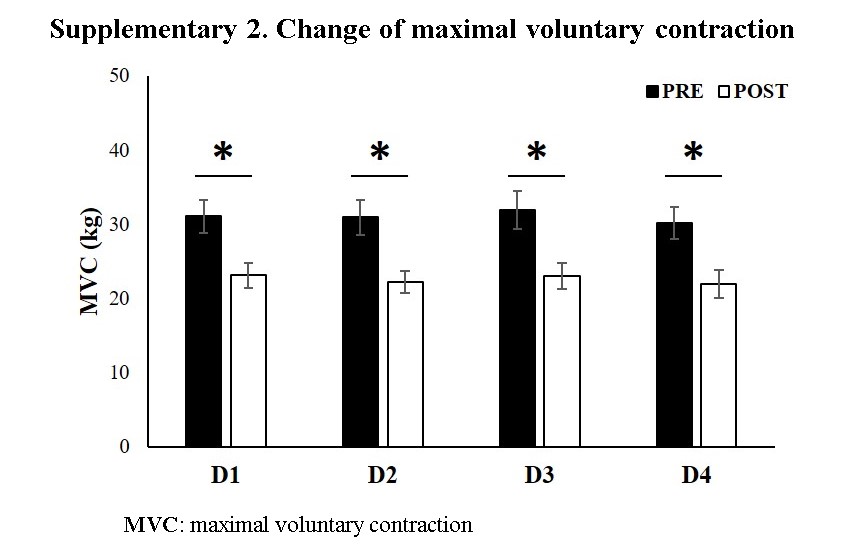

Supplement: Supplementary file 2 — Supplementary file2 (JPG 48 KB) [file 464_2025_12082_MOESM2_ESM.jpg]

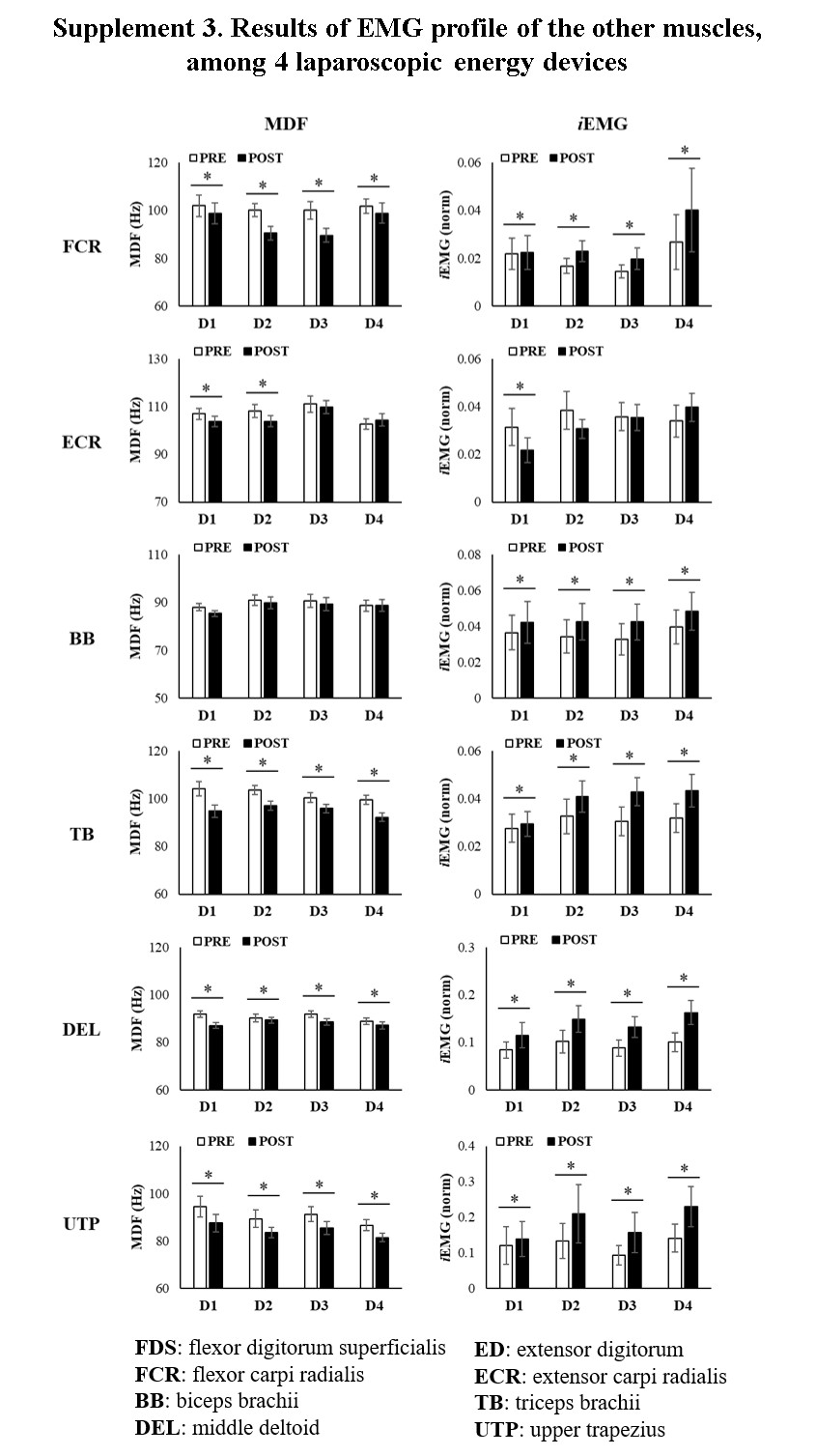

Supplement: Supplementary file 3 — Supplementary file3 (JPG 193 KB) [file 464_2025_12082_MOESM3_ESM.jpg]
